# Maternal psychosocial risk factors and child gestational epigenetic age in a South African birth cohort study

**DOI:** 10.1038/s41398-021-01434-3

**Published:** 2021-07-02

**Authors:** Nastassja Koen, Meaghan J. Jones, Raymond T. Nhapi, Marilyn T. Lake, Kirsten A. Donald, Whitney Barnett, Nadia Hoffman, Julia L. MacIsaac, Alexander M. Morin, David T. S. Lin, Michael S. Kobor, Karestan C. Koenen, Heather J. Zar, Dan J. Stein

**Affiliations:** 1 Department of Psychiatry and Mental Health, University of Cape Town, Cape Town, South Africa; 2 South African Medical Research Council (SAMRC) Unit on Risk and Resilience in Mental Disorders, Cape Town, South Africa; 3 Neuroscience Institute, University of Cape Town, Cape Town, South Africa; 4 Biochemistry and Medical Genetics, Max Rady College of Medicine, Rady Faculty of Health Sciences, University of Manitoba, Children’s Hospital Research Institute of Manitoba, Winnipeg, Manitoba Canada; 5 Department of Paediatrics and Child Health, Red Cross War Memorial Children’s Hospital, University of Cape Town, Cape Town, South Africa; 6 Division of Epidemiology and Biostatistics, School of Public Health and Family Medicine, University of Cape Town, Cape Town, South Africa; 7 Division of Developmental Paediatrics, Department of Paediatrics and Child Health, Red Cross War Memorial Children’s Hospital, University of Cape Town, Cape Town, South Africa; 8 Department of Paediatrics & Child Health and SAMRC Unit on Child and Adolescent Health, University of Cape Town, Cape Town, South Africa; 9 Department of Medical Genetics, Centre for Molecular Medicine and Therapeutics, BC Children’s Hospital Research Institute, University of British Columbia, Vancouver, Canada; 10 BC Children’s Hospital Research Institute, Vancouver, Canada; 11 Canadian Institute for Advanced Research, Toronto, Canada; 12 Department of Epidemiology, Harvard T.H. Chan School of Public Health, Boston, MA USA; 13 Psychiatric and Neurodevelopmental Genetics Unit and Department of Psychiatry, Massachusetts General Hospital, Boston, MA USA

**Keywords:** Epigenetics and plasticity, Epigenetics in the nervous system

## Abstract

Accelerated epigenetic aging relative to chronological age has been found to be associated with higher risk of mortality in adults. However, little is known about whether and how *in utero* exposures might shape child gestational epigenetic age (EA) at birth. We aimed to explore associations between maternal psychosocial risk factors and deviation in child gestational EA at birth (i.e., greater or lower EA relative to chronological age) in a South African birth cohort study—the Drakenstein Child Health Study. Maternal psychosocial risk factors included trauma/stressor exposure; posttraumatic stress disorder (PTSD); depression; psychological distress; and alcohol/tobacco use. Child gestational EA at birth was calculated using an epigenetic clock previously devised for neonates; and gestational EA deviation was calculated as the residuals of the linear model between EA and chronological gestational age. Bivariate linear regression was then used to explore unadjusted associations between maternal/child risk factors and child gestational EA residuals at birth. Thereafter, a multivariable regression method was used to determine adjusted associations. Data from 271 maternal-child dyads were included in the current analysis. In the multivariable regression model, maternal PTSD was significantly and negatively associated with child gestational EA residuals at birth (*β* = −1.95; *p* = 0.018), controlling for study site, sex of the child, head circumference at birth, birthweight, mode of delivery, maternal estimated household income, body mass index (BMI) at enrolment, HIV status, anaemia, psychological distress, and prenatal tobacco or alcohol use. Given the novelty of this preliminary finding, and its potential translational relevance, further studies to delineate underlying biological pathways and to explore clinical implications of EA deviation are warranted.

## Introduction

Maternal exposure to psychosocial risk factors during pregnancy—including traumatic stressors, psychiatric disorders and symptoms, and substance misuse – constitute a notable public health concern; and may be associated with poor birth outcomes and adverse health and development in affected children [1–3]. Given the high burden of psychosocial risk factors in low- and middle-income countries (LMICs) such as South Africa [4], maternal-child dyads in these settings are particularly vulnerable. Prenatal psychosocial risk exposure may be associated with measurable changes in DNA methylation (DNAm) at specific genomic sites in the affected children [5]. It is noteworthy, though, that a recent meta-analysis found no large, epigenome-wide effects of prenatal maternal stress on child differential DNAm [6]. Recent work has also investigated DNAm as a predictor of chronological age in children and adults [7, 8]. However, given the limitations in transferability of such ‘epigenetic clocks’ (which were calibrated initially for adult populations); specialised clocks have recently been generated to estimate gestational age at birth [9–11].

Accelerated epigenetic aging—i.e., greater DNAm gestational age relative to chronological age—has been found to be associated with increased risk of mortality in adults [12–14] (and potentially with increased risk of age-related disorders [14]). Further, recent work has reported significant associations between a number of maternal-child biomedical factors (e.g., pre-pregnancy maternal obesity; maternal age over 40 years at delivery; lower 1 min Apgar score) [15, 16] and accelerated epigenetic aging in the affected children. Of note, associations with lower relative epigenetic age (EA) have also been reported (e.g., in the case of prior insulin-treated gestational diabetes mellitus [15]). Further, there is emerging interest in the potential effects of psychosocial risk factors—for example, maternal depression [17], and greater infant distress (in the context of low caregiver contact) [18] have each been found to be associated with lower EA relative to chronological age. However, there remains a relative paucity of existing literature in this field, particularly in LMICs.

This analysis was nested within the Drakenstein Child Health Study (DCHS), an ongoing, interdisciplinary, longitudinal birth cohort study investigating determinants of maternal-child health in a poor, peri-urban South African community [19–22]. We have previously reported a high burden of maternal psychosocial risk factors (e.g., traumatic stress exposure/posttraumatic stress disorder (PTSD), alcohol and tobacco use); and significant associations between these factors and adverse birth and neurodevelopmental outcomes in the affected children in this cohort [22–27]. In the current analysis, we aimed to investigate associations between maternal psychosocial risk factors and deviation in child gestational EA at birth, i.e., greater or lower EA relative to chronological age. We hypothesised that key maternal risk factors would be significantly associated with our outcome of interest in this study sample.

## Materials/subjects and methods

### Participants

Pregnant women were recruited at 20–28 weeks’ gestation from two primary care clinics in the Drakenstein sub-district in Paarl, Western Cape, South Africa—one (TC Newman) serves a predominantly mixed ancestry community; while the other (Mbekweni) serves primarily a black African ancestry community. Within the DCHS, mothers are followed throughout pregnancy and childbirth until the index child is at least 5 years old [19–22]. General inclusion and exclusion criteria for the DCHS are described fully elsewhere [20].

For this analysis, samples of mothers with complete and accurate sociodemographic, biomedical and psychosocial phenotype data; with recorded maternal informed consent; and with non-contaminated Illumina Infinium HumanMethylation450K or MethylationEPIC BeadChip umbilical cord blood data were eligible for inclusion [28] (see also *Statistical Analyses*, below).

This study sample included mothers enroled into the larger study between March 2012 and March 2015. From a total of 1225 mothers enroled, 1143 were retained in the cohort and delivered live births. From this sub-sample, 275 dyads had non-contaminated HumanMethylation450K (Batch 1, *n* = 120) or MethylationEPIC (Batch 2, *n* = 155) BeadChip cord blood data [28]; and available maternal and child phenotype data. This sub-sample was initially selected for analysis based on a number of risk criteria of relevance to the larger DCHS. Of note, cord blood samples analysed using the EPIC BeadChip (Batch 2) were selected primarily to optimise those with prenatal maternal trauma exposure and/or PTSD. Two sets of twins were excluded, due to concerns of analytic mixed effects. Thus, data from a sub-sample of 271 maternal-child dyads were included in the current analysis.

### Variables and measurement

Maternal sociodemographic characteristics; antenatal biomedical and psychosocial risk factors; and anthropometric outcomes at birth were assessed, as is detailed below. Maternal psychosocial assessment in the DCHS has been described fully previously [20].

#### Maternal sociodemographic characteristics

Socioeconomic status (SES) and related sociodemographic characteristics were assessed using a questionnaire adapted from the South Africa Stress and Health Study (SASH) [29].

#### Maternal biomedical risk factors

For our purposes, maternal Human Immunodeficiency Virus (HIV) status was assessed by rapid testing at enrolment if the mother’s HIV status was unknown at that time. Pre-eclampsia was defined as new-onset hypertension after 20 weeks’ gestation with proteinuria or other organ dysfunction; and maternal anaemia was conservatively defined as any haemoglobin measurement <10 g/dl [22].

#### Maternal antenatal psychosocial risk factors

Exposure to stressful events—either recently or in one’s lifetime—was assessed using the modified World Mental Health Life Events Questionnaire (LEQ; adapted for our purposes from the SASH [29]); the Childhood Trauma Questionnaire (CTQ) [30]; and the Intimate Partner Violence (IPV) Questionnaire (adapted from the World Health Organization (WHO) multi-country study on women’s health and domestic violence against women [31]. The modified PTSD Symptom Scale (mPSS) [32, 33] was used as a rapid screening for PTSD, based on the DSM diagnostic criteria (with additional introductory items devised for our purposes to assess for the presence of a DSM-defined psychological trauma). The mPSS screens for PTSD symptoms in the preceding 2 weeks. In addition—for the purposes of the DCHS—we generated a composite measure of maternal lifetime trauma exposure (as assessed antenatally), incorporating data from the LEQ, CTQ, IPV questionnaire, and mPSS; as well as from the clinician-administered MINI (Mini International Neuropsychiatric Interview) [34–36]. Psychological distress was assessed using the Self-Reporting Questionnaire (SRQ-20) [37–39]; and depression using the Beck Depression Inventory II (BDI-II) [40–42] and the Edinburgh Postnatal Depression Scale (EPDS) [43, 44]. Maternal substance use^1^ was identified using the WHO Alcohol, Smoking, and Substance Involvement Screening Test (ASSIST) [45–47] and a retrospective Alcohol Exposure Questionnaire (AEQ). The AEQ was designed for the purposes of the DCHS to quantify alcohol use (during any of the three trimesters of pregnancy) in mothers assessed as high-risk on the ASSIST.

#### Anthropometry at birth

Newborn weight and head circumference at birth were measured by trained clinical staff, and the relevant z scores were then calculated using the Fenton preterm growth charts [48, 49]. Following the WHO convention, low weight-for-age z score (WAZ) and low head circumference-for-age z score (HCAZ) each was defined as a score of 2 standard deviations or more below the mean [50]. Prematurity was defined as birth before 37 completed weeks’ gestation, as estimated by antenatal ultrasound measurement (for the majority of participants). In the rare case of missing or implausible data, fundal height and then maternal recall of last menstrual period were used to determine gestation at birth. Low birthweight was defined as less than 2500 g.

#### Umbilical cord blood collection

In the DCHS, umbilical cord blood was collected by trained staff after newborn delivery but before delivery of the placenta. The cord was clamped and cut, after which the clamp was released and cord blood drained by gravity into a kidney dish. Thereafter, cord blood was collected using a syringe for processing and storage [28]. Staff were specifically trained to ensure only cord blood drained into the collection dish and that to their best ability, no other/external blood was collected. Further, cord blood samples previously identified as being considerably contaminated with maternal blood (presumably resulting from mixing of blood during sample collection) [28], were excluded from the current analysis.

### Ethical considerations

The DCHS was approved by the Human Research Ethics Committee (HREC) of the Faculty of Health Sciences, University of Cape Town (UCT) and by the Western Cape Provincial Research Committee. Within the DCHS, all potential participants were provided with a consent form which included a description of the scope and aims of the study, in their preferred language (English, Afrikaans, or isiXhosa). Following verbal and written informed consent, mothers were asked to complete a battery of self-report measures, and to respond to select clinician-administered assessment tools (e.g., the MINI) at an antenatal study visit (28–32 weeks’ gestation). While maternal phenotype data from a number of postnatal timepoints are also collected within the larger DCHS [19, 20]; the current analysis pertains to the antenatal maternal assessment only.

All measures were administered by trained study staff in either English, Afrikaans or isiXhosa, as per participant preference. Interviews were conducted in private onsite consultation rooms and every effort was made to ensure confidentiality. Participants were also provided with standard reimbursement for transport costs. On completion of the study assessment, participants with suspected psychopathology or psychosocial risk were referred by study staff to appropriate care providers in the community, according to a standard operating procedure devised for the DCHS. Health promotion information leaflets and bookmarks designed by the study team (and translated into participants’ preferred language) were also offered to all enroled mothers, and included contact details for local and accessible health service providers in addition to the formal referral processes [25].

### Statistical methods

Data were analysed using R [51] and STATA [52].

#### Maternal-child phenotype

Frequency distributions and medians (interquartile ranges) were used to describe maternal sociodemographic characteristics; biomedical and psychosocial risk profiles; as well as infant anthropometric parameters at birth.

#### Gestational epigenetic age at birth

As has been described fully previously [28], cord blood DNAm was measured using the Illumina Infinium HumanMethylation450K (Illumina, San Diego, USA) or the MethylationEPIC BeadChip [53] as per manufacturers’ instructions. Raw data were imported into Illumina GenomeStudio Software for background subtraction and colour correction, and then exported for processing using the *lumi* package in R (version 3.2.3). Quality control procedures were standardised and are discussed in detail elsewhere [28, 54]. In summary, CpGs with high detection p values (>0.05), those on the X or Y chromosome, and those with prior poor performance were removed. Normalisation was undertaken using subset quantile within array (SWAN) for the 450K data; or beta mixture quantile (BMIQ) for the EPIC data. Finally, ComBat was used to correct for chip (all data) and row effects (450K data only).

Gestational EA at birth was calculated using an epigenetic clock particularly designed for newborns which outputs gestational age [11] and is based on prior work in adult populations using chronological age [7]. This gestational age clock [11] was trained on 1068 samples (mean ultrasound estimated gestational age 279.6 days with 95% CI 279–280.3 days) and tested on 685 samples (mean ultrasound estimated gestational age 279.4 days with 95% CI 278.5–280.2 days), all from the Norwegian Mother and Child Cohort Study (MoBa) study [55, 56]. From 5,474 CpGs associated with ultrasound-estimated gestational age, the final elastic net regression predictor contained 96 CpGs, 8 of which are missing on the EPIC array. For samples in this study generated using the EPIC data, the 8 missing CpGs were set to the mean values of the 450K samples. In the current analysis, gestational EA deviation was calculated as the residuals of the linear model between gestational EA and chronological gestational age [57], stratified by batch.

#### Associations between maternal psychosocial risk factors and gestational epigenetic age deviation at birth

Bivariate analyses were used to explore unadjusted associations between key sociodemographic, biomedical and psychosocial variables, Supplementary Table A, and child gestational EA deviation at birth. Thereafter, a multivariable regression approach was used to determine adjusted associations. The multivariable regression model was obtained by retaining variables with at least a trend-level difference (*p* < 0.1) at the bivariate level (in attempts to minimize Type 2 error); and those that theoretically were suspected or hypothesized to be significantly associated with the outcome of interest. Stratification of the regression model by batch/methylation microarray (i.e., Infinium HumanMethylation450K versus MethylationEPIC BeadChip) was not undertaken; as samples analysed using the EPIC BeadChip were selected based primarily on trauma/PTSD phenotype. Given that this was not the case for samples analysed using the 450K chip, selection criterion was a potential confounder. Further, EA was found not to differ significantly between batches in exploratory t-test analyses. Nonetheless, potential batch effects were taken into account by stratifying the primary outcome computations—EA residuals—by batch (as described above).

## Results

### Maternal sociodemographic and biomedical characteristics

This study sample comprised 151 (56%) women recruited from Mbekweni and 120 (44%) from TC Newman. At enrolment, the median (IQR) age of mothers was approximately 26 (22; 31) years, Table 1. Approximately a third of mothers were primigravid, and most (61%) were neither married nor co-habiting with a partner. Despite approximately half of the study sample having completed some secondary education, nearly three quarters (73%) were unemployed and the vast majority (83%) reported a monthly household income of ≤ ZAR 5000 (≈USD 350). The prevalence of maternal HIV infection in the study sample was 24%, which is representative of the prevalence (21%) in the larger DCHS cohort [22]. A statistically significant site-specific difference in maternal HIV infection was also noted (*p* < 0.01), with a significantly higher prevalence at Mbekweni, than at TC Newman. No child in this study sample was HIV infected.

**Table 1:** Maternal sociodemographic characteristics and antenatal psychosocial risk^++^.

Variable	Mbekweni - *n* (%) (99% black African ancestry)	TC Newman – *n* (%) (98% mixed ancestry)	Total - *n* (%)	*P*-value
*Maternal sociodemographic & biomedical characteristics*				
Number of mothers	151 (56)	120 (44)	271 (100)	0.06
Age at enrolment: median (IQR) years	27 (22; 32)	25 (22; 30)	26 (22; 31)	0.07
Primigravida	49 (32)	42 (35)	91 (34)	0.76
Married/cohabiting	54 (36)	52 (43)	106 (39)	0.27
Educational achievement				
Some primary education	10 (7)	11 (9)	21 (8)	0.13
Some secondary education	87 (58)	54 (45)	141 (52)	
Completed secondary education	45 (30)	50 (42)	95 (35)	
Tertiary education	9 (6)	5 (4)	14 (5)	
Employed	33 (22)	39 (33)	72 (27)	0.07
Estimated household income				
< ZAR 1000/month	71 (47)	41 (34)	112 (41)	0.08
ZAR 1000 – ZAR 5000/month	60 (40)	55 (46)	115 (42)	
> ZAR 5000/month	20 (13)	24 (20)	44 (16)	
HIV-infected	58 (38)	6 (5)	64 (24)	<0.01
*Maternal antenatal psychosocial risk*				
Lifetime trauma exposure (mPSS/LEQ)	107 (71)	63 (53)	170 (63)	<0.01
Lifetime trauma exposure (composite)	137 (91)	101 (84)	238 (88)	0.07
Childhood maltreatment (CTQ): above threshold	52 (34)	61 (51)	113 (42)	0.01
Intimate Partner Violence (IPV) exposure				
Any lifetime exposure	69 (46)	76 (63)	145 (54)	0.01
Any recent exposure	56 (37)	59 (49)	115 (42)	0.07
Stressful life events: median (IQR) score	1 (1; 3)	3 (1; 4)	2 (1; 3)	<0.01
Psychological distress (SRQ-20): above threshold	39 (26)	39 (33)	78 (29)	0.27
Current PTSD (mPSS)	63 (42)	19 (16)	82 (30)	<0.01
Depression (BDI-II): above threshold	34 (23)	33 (28)	67 (25)	0.47
Depression (EPDS): above threshold	42 (28)	40 (33)	82 (30)	0.41
Alcohol exposure during pregnancy	21 (14)	25 (21)	46 (17)	0.20
Any tobacco use during pregnancy (ASSIST)	14 (9)	64 (53)	78 (29)	<0.01

^++^Prevalence computed including missing data.

### Maternal antenatal psychosocial risk

Most mothers (63%) in this study sample reported exposure to any psychological trauma during their lifetimes, as assessed using the mPSS and LEQ. When applying our composite trauma exposure variable, this prevalence was found to increase to 88%, Table 1. Almost half (42%) of the study sample had been exposed to maltreatment (abuse/neglect) during childhood; and the prevalence of lifetime exposure to intimate partner violence (IPV) was 54% (with 42% having experienced IPV during the past year). The prevalence of current PTSD was 30%, with significantly more mothers at Mbekweni than TC Newman affected.

Despite a relatively low median (IQR) score on the Life Events Questionnaire [2 (1; 3)], 29% of the study sample reported symptoms of psychological distress on the SRQ-20. Similarly, a quarter scored above threshold for depression on the BDI-II (25%) (and almost a third on the EPDS (30%)). Alcohol consumption during pregnancy was reported in 17% of participants, and prenatal tobacco use in 29%. A notable site-specific difference was evident, with the prevalence of tobacco use significantly higher among mothers from TC Newman.

### Anthropometry at birth

More than half (55%) of children in this study sample was male; and the median (IQR) gestational age at birth was 39 (38; 40) weeks, Table 2. Approximately a fifth (21%) of the index deliveries were by Caesarean section. The prevalence each of preterm birth and of low birth weight was 11%; and of decreased weight-for-age z (WAZ) scores at birth and low head-circumference-for-age z (HCAZ) scores, 8% and 13%, respectively. The median (IQR) weight at birth in this study sample was 3.1 (2.8; 3.5) kg; and the median (IQR) head circumference was 34 (33; 35) cm.

**Table 2:** Anthropometry at birth^++^.

Variable	Mbekweni - *n* (%)	TC Newman - *n* (%)	Total - *n* (%)	*P*-value
Sex of child				
Male	81 (54)	69 (58)	150 (55)	0.61
Female	70 (46)	51 (43)	121 (45)	
Gestational age at birth: median (IQR) weeks	39 (38; 40)	39 (38; 40)	39 (38; 40)	0.79
Preterm birth	18 (12)	12 (10)	30 (11)	0.76
Birth by Caesarean section	37 (25)	20 (17)	57 (21)	0.16
Birthweight: median (IQR) kg	3.2 (2.9; 3.5)	3.0 (2.7; 3.4)	3.1 (2.8; 3.5)	0.01
Low birthweight: < 2500 g	14 (9)	15 (13)	29 (11)	0.54
Birth WAZ: median (IQR)	−0.4 (−1.1; 0.2)	−0.7 (−1.4; −0.1)	−0.5 (−1.3; −0.01)	0.03
Low WAZ at birth: ≤ 2 SDs below mean weight-for-age	11 (7)	11 (9)	22 (8)	0.76
Head circumference at birth: median (IQR) cm	34 (33; 35)	34 (32; 35)	34 (33; 35)	0.06
Birth HCAZ: median (IQR)	−0.5 (−1.2; 0.4)	−0.6 (−1.3; 0.2)	−0.6 (−1.2; 0.4)	0.23
Low HCAZ at birth: ≤ 2 SDs below mean head-circumference-for-age	20 (13)	14 (12)	34 (13)	0.77

^++^Prevalence computed including missing data.

### Associations between maternal psychosocial risk and gestational epigenetic age deviation at birth

In bivariate unadjusted analyses, mode of delivery (*β* = 1.87; *p* = 0.026), newborn head circumference at birth (*β* = 0.62; *p* = 0.001), birth weight (*β* = 1.97; *p* = 0.002) and weight-for-age z-score at birth (*β* = 0.84; *p* = 0.012), each was found to be significantly positively associated with gestational EA residuals at birth, using the conventional *p*-value < 0.05 cut-off. When accounting for potential trend-level relationships (*p* < 0.1), positive associations with the outcomes of interest were also noted for estimated household monthly income of less that ZAR 1000 (≈USD 70) (*β* = 1.35; *p* = 0.052), maternal body mass index (BMI; kg/m^2^) at enrolment (*β* = 0.10; *p* = 0.068) and maternal anaemia (*β* = 1.60; *p* = 0.091).

Maternal psychological distress (*β* = −1.57; *p* = 0.038) and low infant birthweight (*β* = −2.33; *p* = 0.035) were found to be significantly negatively associated with gestational EA residuals at birth; while maternal HIV (*β* = −1.35; 0.094) and PTSD (*β* = −1.24; *p* = 0.096) showed trend-level negative associations (Table 3, Supplementary Table A).

**Table 3:** Associations between maternal psychosocial risk factors and gestational epigenetic age deviation at birth.

	Unadjusted β estimate [95% CI]	*P*-value	Adjusted β estimate [95% CI]	*P*-value
Study site: Mbekweni	0.83 [−0.53; 2.18]	0.231	0.83 [−1.04; 2.70]	0.382
Estimated household income: < ZAR 1000/month	1.35 [−0.01; 2.71]	0.052^b^	1.87 [0.40; 3.34]	0.013^a^
Maternal BMI^c^ at enrolment	0.10 [−0.01; 0.21]	0.068^b^	0.07 [−0.04; 0.19]	0.217
Mother HIV-infected	−1.35 [−2.93; 0.23]	0.094^b^	−1.87 [−3.74; −0.01]	0.049^a^
Maternal anaemia	1.60 [−0.26; 3.47]	0.091^b^	1.70 [−0.24; 3.64]	0.085^b^
Mode of delivery: Caesarean section	1.87 [0.23; 3.51]	0.026^a^	1.63 [−0.20; 3.47]	0.081^b^
Sex of child: Male	−0.59 [−1.94; 0.77]	0.396	−1.17 [−2.62; 0.28]	0.113
Child head circumference at birth	0.62 [0.25; 0.98]	0.001^a^	0.20 [−0.34; 0.74]	0.469
Child weight at birth	1.97 [0.73; 3.20]	0.002^a^	1.62 [−0.13; 3.37]	0.069^b^
Alcohol exposure during pregnancy	−0.13 [−1.91; 1.65]	0.885	0.13 [−1.92; 2.17]	0.904
Tobacco use during pregnancy	−0.76 [−2.23; 0.72]	0.313	−0.20 [−2.19; 1.79]	0.843
Psychological distress	−1.57 [−3.04; −0.09]	0.038^a^	−0.76 [−2.40; 0.88]	0.363
Current PTSD	−1.24 [−2.69; 0.22]	0.096^b^	−1.95 [−3.57; −0.33]	0.018^a^

^a^*p* < 0.05.

^b^*p* < 0.1.

^c^BMI (Body Mass Index): kg/m^2^.

In the final multivariable regression model, maternal PTSD was significantly and negatively associated with gestational EA residuals at birth (*β* = −1.95; *p* = 0.018), when controlling for study site, sex of the child, head circumference at birth, birthweight, mode of delivery, maternal estimated household income, BMI at enrolment, HIV status, anaemia, psychological distress, prenatal tobacco use and alcohol consumption, Table 3.

## Discussion

In this study of the association between maternal exposure to prenatal psychosocial risk factors and gestational epigenetic age (EA) deviation at birth in the index children, we found a negative association with EA residuals in children born to mothers with PTSD (compared to those without such exposure), when controlling for a number of relevant covariates.

These findings are comparable to emerging—albeit mixed—evidence of trauma-/PTSD-associated EA deviation in adult combat veterans. For example, recent work has demonstrated a positive association each between lifetime PTSD severity [58] and PTSD hyperarousal symptoms [59], and EA residualized for chronological age in male and female veterans (although the association with PTSD severity was not replicated in the latter study [59]). Further, a meta-analysis by the Psychiatric Genomics Consortium PTSD Epigenetics Workgroup across a number of adult cohorts reported small but significant associations each between childhood trauma exposure and severity of lifetime PTSD, and accelerated epigenetic aging (positive EA residuals) [60]. However, neither current PTSD diagnosis/severity nor lifetime trauma exposure was associated with this outcome. In addition, a small longitudinal study of male soldiers (*n* = 96), assessing pre- and post-deployment EA reported that the development of PTSD symptoms was associated with a relative decrease in EA over time (although trauma was found to be significantly associated with an increase in EA in this study) [61].

Work in paediatric study samples has yielded similarly varied findings. For example, one recent study (*N* = 101) reported a positive correlation between age acceleration (measured as the residual between EA and chronological age) and direct exposure to neighbourhood violence in children aged 6–13 years [62]; though—as discussed above—a negative association between distress and EA deviation has also been reported [18]. Thus, it may be that any deviation in EA (i.e., greater or lower EA relative to chronological age) is associated with exposure to prenatal maternal risk [15, 17]; and that the functional relevance and implications of accelerated epigenetic aging in infants and children are distinct from those previously demonstrated in adult study samples [12–14].

A number of limitations of the current study should be acknowledged. First, the sample size was relatively small, thus decreasing the statistical power of association analyses. Nevertheless, we report an important association with PTSD exposure even after taking into account study site, sex of the child, head circumference at birth, birthweight, mode of delivery, maternal estimated household income, BMI at enrolment, HIV status, anaemia, psychological distress, and prenatal tobacco or alcohol use. Second, self-report questionnaires to assess maternal exposure to psychosocial risk factors may have resulted in under-reporting of key variables such as substance use. Given prior evidence of associations between maternal smoking and alcohol consumption and deviations in EA [63], self-reporting bias may have contributed to the lack of significant associations in our analysis. However, we have previously shown that self-reported tobacco smoking correlated well with urine cotinine measurement (an objective biomarker of tobacco exposure) in our study cohort; particularly in the TC Newman sample [27]. Third, PTSD was not assessed using a clinician-administered diagnostic interview, which may have biased the findings. Further, heterogenous and dynamic definitions of trauma exposure and PTSD applied in the field may also limit the interpretation and generalisability of our current findings. For example, investigators may elect a “strict” approach using the DSM 5 Criterion A definition for trauma exposure [64], versus adopting a more inclusive approach, e.g., incorporating subjectively stressful events which do not necessarily meet the DSM criteria (as we have done in the current analysis). Similarly, “broad” versus “narrow” definitions of PTSD [65] would also impact estimations of case prevalence; and our findings were not replicated with more stringent definitions of trauma/PTSD.

Fourth, given that samples for analysis using the EPIC array were initially selected primarily based on maternal trauma or PTSD phenotype, the multivariable regression models could not be further stratified by chip or batch, due to the confounding effect of our selection criteria. We did, however, attempt to address potential batch effects by stratifying the age residuals by batch. Given the noted site-specific differences in maternal PTSD and other key phenotypes (e.g., tobacco use), we also included study site as a covariate in the multivariable regression model. Fifth, gestational age estimation (including by ultrasound) may be prone to notable measurement error [66–68], which may in turn have biased our primary outcome variable (gestational EA residuals at birth). Finally, inconsistencies between our results and the null findings of the well-powered epigenome-wide meta-analysis of maternal stress [6] (discussed in the Introduction, above) may be accounted for by differences in analytic approaches (e.g., differential DNAm versus EA deviation analysis); in microarray chip used (only 450K data were included in the meta-analysis); in sample size; or in phenotype variables (e.g., ancestral diversity in our study sample, differing prevalence of prenatal maternal smoking and variable definitions of stress, trauma and PTSD).

These limitations notwithstanding, our study provides a unique preliminary exploration of associations between maternal psychosocial risk factors and child gestational EA deviation at birth in a high-risk LMIC environment. To the best of our knowledge, an association between maternal PTSD and gestational EA deviation in the index children has not previously been reported. Future studies—incorporating larger samples sizes and cross-tissue analyses—are warranted in order to explore such associations further; and to delineate underlying neurobiological mechanisms. In addition, work is needed to understand the implications of EA deviation at birth. For example, it is currently unknown whether and how EA deviation may also be associated with adverse neurodevelopmental outcomes in the affected children, although interest in this field is growing [17]. Further, while the adverse transgenerational effects of maternal trauma exposure and PTSD have been well documented—both in the DCHS cohort [23–25] and globally [1, 69]—the potential role of EA deviation in these associations is yet to be determined. From a translational perspective, if EA deviation at birth does indeed prove clinically meaningful, it may contribute to an improved neurobiological understanding of transgenerational trauma and PTSD, which may in turn inform the development of epigenetic biomarkers in this context.

## Supplementary information

Supplementary Table A

## Footnotes

1 Prenatal tobacco use derived from ASSIST item assessing recent frequency of use.
