# Supplementary material for: Maternal psychosocial risk factors and child gestational epigenetic age in a South African birth cohort study
Source: Transl Psychiatry. 2021 Jul 2;11:358. doi: 10.1038/s41398-021-01434-3 (PMC8253754; doi:10.1038/s41398-021-01434-3)
Supplement: Supplementary file 1 — Supplementary Table A [file 41398_2021_1434_MOESM1_ESM.pdf]

SUPPLEMENTARY TABLE A: Unadjusted associations between maternal & child characteristics, and gestational epigenetic age deviation at birth

	Unadjusted β Estimate [95% CI]	P-Value
**CORE VARIABLES**		
Study site: Mbekweni	0.83 [-0.53; 2.18]	0.231
Sex of child: Male	-0.59 [-1.94; 0.77]	0.396
Mother HIV-infected	-1.35 [-2.93; 0.23]	0.094*
Alcohol exposure during pregnancy	-0.13 [-1.91; 1.65]	0.885
Tobacco use during pregnancy	-0.76 [-2.23; 0.72]	0.313
**MATERNAL SOCIODEMOGRAPHIC CHARACTERISTICS**		
Age at enrolment	-0.06 [-0.18; 0.06]	0.329
Marital status: married/cohabiting	0.36 [-0.99; 1.71]	0.600
Estimated household income: < ZAR 1000/month	1.35 [-0.01; 2.71]	0.052*
Educational achievement: Primary or Some Secondary	-0.52 [-1.90; 0.86]	0.457
**MATERNAL ANTENATAL BIOMEDICAL CHARACTERISTICS**		
BMI+ at enrolment	0.10 [-0.01; 0.21]	0.068*
Anaemia	1.60 [-0.26; 3.47]	0.091*
Pre-eclampsia	-1.11 [-4.88; 2.65]	0.561
**MATERNAL ANTENATAL PSYCHOSOCIAL RISK**		
Lifetime trauma exposure (mPSS/LEQ)	-0.88 [-2.27; 0.50]	0.212
Lifetime trauma exposure (composite)	1.12 [-0.97; 3.22]	0.292
Childhood maltreatment (CTQ): Above threshold	0.10 [-1.26; 1.46]	0.883
Intimate Partner Violence (IPV) exposure		
Any lifetime exposure: Above threshold	0.11 [-1.23; 1.46]	0.869
Any recent exposure: Above threshold	0.35 [-1.00; 1.71]	0.608
Stressful life events	-0.05 [-0.39; 0.29]	0.776
Psychological distress (SRQ): Above threshold	-1.57 [-3.04; -0.09]	0.038**
Current PTSD (mPSS)	-1.24 [-2.69; 0.22]	0.096*
Depression (BDI-II): Above threshold	-0.02 [-1.58; 1.54]	0.981
Depression (EPDS): Above threshold	-1.00 [-2.45; 0.46]	0.178
**CHILD BIOMEDICAL CHARACTERISTICS AT BITH**		
Mode of delivery: Caesarean section	1.87 [0.23; 3.51]	0.026**
Preterm birth	-0.87 [-3.02; 1.28]	0.428
Apgar score: 1 min	-0.30 [-0.93; 0.32]	0.343
Apgar score: 5 min	0.90 [-0.66; 2.46]	0.256
**CHILD ANTHROPOMETRY AT BIRTH**		
Birthweight	1.97 [0.73; 3.20]	0.002**
Low birthweight	-2.33 [-4.49; -0.16]	0.035**
Birth WAZ	0.84 [0.19; 1.49]	0.012**
Low WAZ at birth	-1.23 [-3.69; 1.24]	0.328
Head circumference at birth	0.62 [0.25; 0.98]	0.001**
Birth HCAZ	0.22 [-0.19; 0.63]	0.285
Low HCAZ at birth	-0.30 [-2.34; 1.74]	0.770

**p<0.05

*p<0.1

+BMI (Body Mass Index): kg/m^2
